# Audit on Antidepressant Prescribing; Documentation of Indication; and Compliance With EIPN Standards in Medication Review, at Hailsham Early Intervention in Psychosis Service

**DOI:** 10.1192/bjo.2024.556

**Published:** 2024-08-01

**Authors:** Daniel Di Francesco, James Todd, Alice Brooke

**Affiliations:** Sussex Partnership NHS Foundation Trust, Hailsham, United Kingdom

## Abstract

**Aims:**

- To improve documentation of antidepressant prescribing in our service, aiming to improve frequency of review, and guide measurement of outcomes.
- Identify patients requiring medical review.

The standards that we audited against are that, for patients under The Early Intervention in Psychosis Service (EIPS), a diagnosis should be recorded alongside each antidepressant prescription and, according to EIPN guidelines, psychotropic medications should be reviewed every 6 months.

Population data from the UK indicates that lack of recording of a diagnosis is associated with increased duration of treatment, and reduced frequency of mental health reviews.

**Methods:**

It was recorded for each patient whether they had an antidepressant prescribed, which medication, the documented indication, and their most recent medical review. Data was collected in a ‘snapshot' cross section of all 89 patients on the caseload in December 2023.

Data was obtained from carenotes by reviewing clinic letters and clinical notes; and cross-referencing with GP records.

**Results:**

33 patients (37%) were prescribed an antidepressant. Of these, 25 (76%) had a recorded indication. The commonest indication was mixed anxiety and depression followed by depression. Sertraline was by far the commonest prescribed antidepressant (52%) followed by mirtazapine. 3 patients were prescribed combination antidepressants. 67 patients (84%) had had a medical review within 6 months.

**Conclusion:**

Among patients with a first episode of psychosis, there is a significant comorbidity of depression and anxiety spectrum disorders.

Our standard was met for most patients but there were several exceptions, and we considered why 8 patients did not have a listed diagnosis. There can be a degree of diagnostic uncertainty in distinguishing anxiety and depressive disorders from negative symptoms, and the affective changes that are an established part of recovery from an acute psychotic episode. In these circumstances it may be appropriate to consider a trial of antidepressants in consultation with the patient. Some of these patients also have been on long-term therapy which preceded their referral to EIPS, leading to uncertainty of the indication and pre-morbid status.

We conclude the following recommendations:

1. Prompt a review of antidepressant use in those identified without a clear indication, discussing risks and benefits with the patient at next review.
2. Arrange medical reviews for those exceeding the 6-monthly window.
3. Record last review for patients under shared care.
4. Re-audit in 6 months to monitor improvement.

